# Cardiac decompensation of patients before transcatheter aortic valve implantation—clinical presentation, responsiveness to associated medication, and prognosis

**DOI:** 10.3389/fcvm.2023.1232054

**Published:** 2023-10-23

**Authors:** Ulrich Fischer-Rasokat, Matthias Renker, Efstratios I. Charitos, Christopher Strunk, Julia Treiber, Andreas Rolf, Maren Weferling, Yeong-Hoon Choi, Christian W. Hamm, Won-Keun Kim

**Affiliations:** ^1^ Department of Cardiology, Kerckhoff Heart Center, Bad Nauheim, Germany; ^2^ Department of Cardiac Surgery, Kerckhoff Heart Center, Bad Nauheim, Germany; ^3^ German Center for Cardiovascular Research (DZHK), Partner Site Rhein-Main, Bad Nauheim, Germany; ^4^ Medical Clinic I (Cardiology and Angiology), University Hospital of Giessen, Giessen, Germany

**Keywords:** transcatheter aortic valve implantation (TAVI), aortic stenosis, heart failure, cardiac decompensation, angiotensin-converting enzyme inhibitors, angiotensin receptor blockers, prognosis

## Abstract

**Aims:**

Cardiac decompensation (CD) in patients with aortic stenosis is a “red flag” for future adverse events. We classified patients undergoing transcatheter aortic valve implantation (TAVI) into those with acute, prior, or no prior CD at the timepoint of TAVI and compared their clinical presentation, prognosis, and effects of the prescribed medication during follow-up.

**Methods:**

Retrospective analysis of patients of one center fulfilling the criteria of 30-day device success after transfemoral TAVI.

**Results:**

From those patients with no CD (*n* = 1,985) ranging to those with prior CD (*n* = 497) and to those with acute CD (*n* = 87), we observed a stepwise increase in the proportion of patients in poor clinical condition, NYHA class III/IV, low psoas muscle area, fluid overload (rales, oedema, pleural effusion), reduced ejection fraction, renal insufficiency, and anemia. More diuretics but less renin-angiotensin system inhibitors (ACEI/ARB) were prescribed for patients with acute CD compared to other groups. Prior CD (hazard ratio and 95% CI 1.40; 1.02–1.91) and acute CD (1.72; 1.01–2.91), a reduced general condition (1.53; 1.06–2.20), fluid overload (1.54;1.14–2.08), atrial fibrillation (1.76; 1.32–2.33), and anemia (1.43;1.08–1.89) emerged as strong independent predictors of one-year mortality. In all three classes of CD, prescribing of ACEI/ARB was associated with a substantial improvement of survival.

**Conclusions:**

The clinical presentation of (acute or prior) cardiac decompensation in patients with AS overlapped substantially with that of patients with classical signs of heart failure. Our results may support an early treatment strategy in patients with left ventricular dysfuntion before clinical signs of congestion are manifest. Moreover, these patients require intensive medical attention after TAVI.

## Introduction

A history of cardiac decompensation (CD) in patients with aortic stenosis (AS), due to increased afterload or myocardial malfunction, is one of the strongest predictors of outcomes, even after correction of afterload by surgical or transcatheter aortic valve implantation (TAVI) (1–5). The prompt treatment of severe AS in patients with acute CD may improve short-term outcomes and reduce hospital stay and treatment costs (6). While clinical signs and symptoms of patients with acute CD usually move clinicians towards a rapid diagnosis and treatment, it may be more challenging to identify and to interpret signs of cardiac congestion in patients with AS but without acute CD. Moreover, it remains unclear whether and to what extent the clinical presentation of decompensated patients with AS differs from that of patients with heart failure (HF) without concomitant AS and how these characteristics may impact further outcomes in the midterm after successful TAVI. We aimed to describe the clinical characteristics of patients with AS with or without a history of CD, determine their prognostic factors, and correlate clinical outcomes with the prescribed medication at hospital discharge.

## Methods

### Study design, participants, and setting

Patients undergoing transfemoral TAVI for symptomatic, severe AS (aortic valve area index <0.6 cm^2^/m^2^ or mean pressure gradient ≥40 mmHg) at a single, high-volume center were included consecutively in this retrospective, observational registry from January 2011 until January 2021. Only patients who were discharged from hospital with documented medication and who fulfilled the criteria of device success at 30 days post-TAVI according to the Valve Academic Research Consortium-3 (VARC-3) document (7) (freedom from mortality, surgery, or repeated device-related interventions, with intended performance of the valve) were included. Follow-up examinations were scheduled at 3 months (on an outpatient basis) followed by a one-year telephone follow-up call. Follow-up data were obtained from outpatient examinations, telephone interviews, or medical records from referring physicians.

Due to the retrospective nature of this study, a waiver of written informed consent was issued by the local ethics committee (University of Giessen), which approved the study protocol. The study complied with the Declaration of Helsinki.

### Clinical parameters of interest

Medical reports of patients were screened for a history of CD, which was defined as an event of hospitalization due to fluid overload and accompanying dyspnea as documented in the medical history of the patient or as reported by the patient at the time of admission. Patients were then classified into one of three groups: no CD (no history of CD and patients never reported CD), prior CD (prior CD was documented, with timespan in months between last episode of CD and hospital admission for TAVI, or patients reported at least one episode of CD prior to admission, but presented in a stable and clinically compensated condition at admission), or acute CD (patients were referred to the hospital while suffering an episode of acute CD for urgent TAVI). Patients’ clinical condition (age-appropriate, reduced, or poor) and their signs (moist pulmonary rales, peripheral edema) and symptoms (NYHA classification) of cardiac congestion at the timepoint of hospital admission were documented. Type and dosage of the discharge medication after TAVI [angiotensin converting enzyme inhibitors or angiotensin receptor blockrs (ACEI/ARB), beta-blockers, mineralocorticoid antagonist, and statins] were assessed as described earlier (8). Fluid overload was defined in our study as the presence of any documented sign of congestion (pulmonary rales, peripheral edema, pleural effusion).

### Computer tomographic parameters

Computed tomographic (CT) analysis was conducted retrospectively using imaging scans indicated for the planning of the TAVI procedure. Data analyses were performed on a dedicated workstation (syngo.via, Siemens Healthineers, Forchheim, Germany). The psoas muscle area (PMA) was measured in a single post-contrast axial slice using the freehand drawing tool. The slice was chosen at the level of L3 vertebrae in a way such that the lateral tips of both transverse processes were visible for better reproducibility. The left and right psoas muscle areas were carefully measured according to the pure geometrical anatomical boundaries. The areas of the left and right psoas muscle were added and the sum (in cm^2^) was indexed to the body surface area. The resulting PMAi was grouped in tertiles for women and men separately, resulting in sex-specific PMAi tertiles. CT scans were also screened for the presence of pleural effusion.

### Outcome variables

The primary endpoint was death from any cause within one year post-intervention. Secondary endpoints were cardiovascular death according to the VARC-3 consensus document (7) and the combination of cardiovascular death and cardiac decompensation from the timepoint of discharge to one year after TAVI.

### Statistical analysis

Continuous data are reported as median and interquartile range (IQR) and were compared by the Mann–Whitney Kruskal–Wallis test; categorical variables are reported as number and percent and were compared by the *χ*^2^ test. Survival curves were constructed using Kaplan–Meier estimates and were compared by the log-rank test. To account for covariates, univariate Cox proportional hazard models were used to calculate hazard ratios (HR). Variables with a statistically significant effect on the univariate analysis (*p* ≤ 0.01) were entered into a multivariable Cox proportional hazards model and underwent a stepwise bidirectional variable selection process. The following variables were included: body mass index, diabetes, atrial fibrillation, peripheral artery disease, Euroscore II, NYHA class III or IV, general condition, prior cardiac decompensation, fluid overload, glomerular filtration rate, anemia, ejection fraction <40%, mean transvalvular aortic pressure gradient <40%, moderate mitral or tricuspid regurgitation, indexed psoas muscle area tertiles, ACEI/ARB, statins. To appreciate the effect sizes of the predictors as well as the baseline hazard of the final Cox propotional hazards regression model, predictions from the model using clinically relevant prototypical values were constructed. All statistical analyses were performed using the SPSS statistical package version 26 (IBM Corp., Armonk, NY, USA) and R version 4.2.2 [R Core Team (2022). R: A language and environment for statistical computing. R Foundation for Statistical Computing, Vienna, Austria. URL https://www.R-project.org/.]

## Results

In this study population, 1,985 patients were assigned to the group that never experienced a CD (no CD), 497 patients were classified to have experienced at least one CD before hospital admission (prior CD), and 87 patients were diagnosed with a CD at the timepoint of hospital admission (acute CD). Patients with acute CD compared with those with prior CD or no CD had a higher prevalence of atrial fibrillation, anemia, chronic obstructive pulmonary disease, a worse renal function, and a higher calculated risk according to the EuroScore II (Table 1). They presented in a considerably worse clinical condition with significantly more symptoms and signs of fluid overload on clinical examination (pulmonary rales, peripheral edema) and exhibited most frequently the presence of pleural effusion as detected by CT. The ejection fraction was lowest in patients with acute CD, and these patients showed the highest rates of low-gradient aortic stenosis and significant atrio-ventricular valve regurgitation. The proportion of patients in the lowest sex-specific tertile of PMAi was highest among those with acute CD, indicating the highest prevalence of sarcopenia among this patient cohort. Slightly more patients with acute CD were treated with balloon-expandable valves.

**Table 1:** Patient characteristics.

	No CD	Prior CD	Acute CD	*P*-Value
	*n* = 1,985	*n* = 497	*n* = 87	
Demographic data				
Female	1,009 (50.8)	281 (56.5)	41 (47.1)	0.050
Age, year	81.9 (78.6–84.9)	82.5 (78.8–85.9)	80.8 (77.3–84.7)	0.032
BMI, kg/m^2^	27.0 (24.3–30.4)	26.8 (24.0–30.5)	27.1 (23.8–32.2)	0.634
Cardiovascular and pulmonary disease				
Arterial hypertension	1,796 (90.5)	454 (91.3)	73 (83.9)	0.092
Diabetes mellitus	624 (31.4)	204 (41.0)	33 (37.9)	<0.001
CAD	1,202 (60.6)	297 (59.8)	51 (58.6)	0.898
Prior MI	227 (11.4)	73 (14.7)	10 (11.5)	0.136
Atrial fibrillation	740 (37.3)	272 (54.8)	55 (63.2)	<0.001
Prior stroke	262 (13.2)	56 (11.3)	8 (9.2)	0.312
Peripheral artery disease	225 (11.3)	59 (11.9)	14 (16.1)	0.390
EuroScore II	2.80 (2.01–4.32)	3.91 (2.69–5.77)	5.20 (3.59–7.99)	<0.001
ICD at discharge	32 (1.6)	23 (4.6)	10 (11.5)	<0.001
COPD	333 (16.8)	111 (22.3)	20 (23.0)	0.008
Clinical data				
NYHA class III/IV	1,436 (72.3)	439 (88.3)	81 (93.1)	<0.001
Reduced general condition	1,193/1,952 (61.1)	370/494 (74.9)	62/86 (72.1)	<0.001
Poor general condition	21/1,952 (1.1)	20/494 (4.0)	23/86 (26.7)	
Frailty	1,034 (52.1)	312 (62.8)	50 (57.5)	<0.001
Moist rales	43/1,947 (2.2)	42/494 (8.5)	26/86 (30.2)	<0.001
Peripheral oedema	323/1,936 (16.7)	144/489 (29.4)	53/84 (63.1)	<0.001
Any congestion	499/1,817 (27.5)	250/464 (53.9)	72/84 (85.7)	<0.001
Laboratory data				
GFR, ml/min/1.73 m^2^	70 (51–89)	55 (41–75)	46 (34–74)	<0.001
Anemia	588/1,917 (30.7)	198/486 (40.7)	44/85 (51.8)	<0.001
Echocardiographic data				
Ejection fraction, %	65 (58–65)	60 (45–65)	50 (35–62)	<0.001
Ejection fraction ≤40%	125/1,917 (6.5)	79/486 (16.3)	26/85 (30.6)	<0.001
MPG <40 mmHg	677/1,917 (35.3)	228/486 (46.9)	54/85 (63.5)	<0.001
Moderate MR or TR	271 (13.7)	129 (20.0)	34 (39.1)	<0.001
CT data				
Pleural effusion	221/1,830 (12.1)	147/458 (32.1)	47/81 (58.0)	<0.001
PMAi, cm^2^/m^2^	8.19 (6.92–9.85)	7.88 (6.68–9.50)	7.97 (6.61–9.35)	<0.001
Lowest PMAi tertile	572/1,795 (31.9)	159/146 (34.9)	39/81 (48.1)	0.031
Procedural data				
Balloon-expandable valve	720 (36.3)	181 (36.4)	43 (49.4)	0.044

The percentage of patients with prescribed ACEI/ARB at discharge declined significantly when moving from those with no CD to those with acute CD, and even in those patients with acute CD treated by ACEI/ARB, the proportion of patients with a target dose ≥50% was half that of patients with no CD (Table 2). In contrast, beta-blockers, mineralocorticoid antagonists, and loop diuretics were more frequently prescribed in patients with acute CD. The prescribing of statins was slightly less frequent in patients with acute CD compared with the other groups.

**Table 2:** Discharge medication.

	No CD	Prior CD	Acute CD	*P*-Value
	*n* = 1,985	*n* = 496	*n* = 87	
ACEI/ARB	1,591 (80.2)	373 (75.2)	57 (65.5)	<0.001
≥50% target dose	1,042 (52.5)	217 (43.8)	22 (25.3)	<0.001
Beta-blockers	1,423 (71.7)	425 (85.7)	73 (83.9)	<0.001
≥50% target dose	748 (37.7)	231 (46.6)	41 (47.1)	<0.001
MR antagonists	289 (14.6)	136 (27.4)	40 (46.0)	<0.001
≥50% target dose	260 (13.1)	121 (24.3)	34 (39.1)	<0.001
Loop diuretics	1,040 (52.4)	427 (86.1)	84 (96.6)	<0.001
Statins	1,445 (72.8)	327 (65.9)	55 (63.2)	0.003
High-intensity statins	171 (8.6)	50 (10.1)	11 (12.6)	0.002

Data shown as number (%). ACEI/ARB, angiotensin converting enzyme inhibitors/angiogtensin receptor antagonists; MR antagonists, mineralocorticoid antagonists.

We further analyzed the patient group with prior CD. In this group, information on the timespan between the last CD and admission as well as information on fluid overload was available for 379 patients. The timespan ranged from 1 to 108 months: 190 patients (50.1%) reported the last CD 1 month before admission; 154 patients (40.6%) reported the last CD 2–6 months before admission; 35 patients (9.2%) reported the last CD >6 months before the last CD. Manifest fluid overload was documented in 117 (61.6%), 74 (48.1%), and 19 (54.3%) of patients with a timespan of 1, 2–6, or >6 months, respectively (0.042), which was mainly due to the presence of pleural effusion in 67/181 (37.0%), 47/153 (30.7%), and 5/34 (14.7%) patients, respectively (*p* = 0.033).

**Figure 1:**
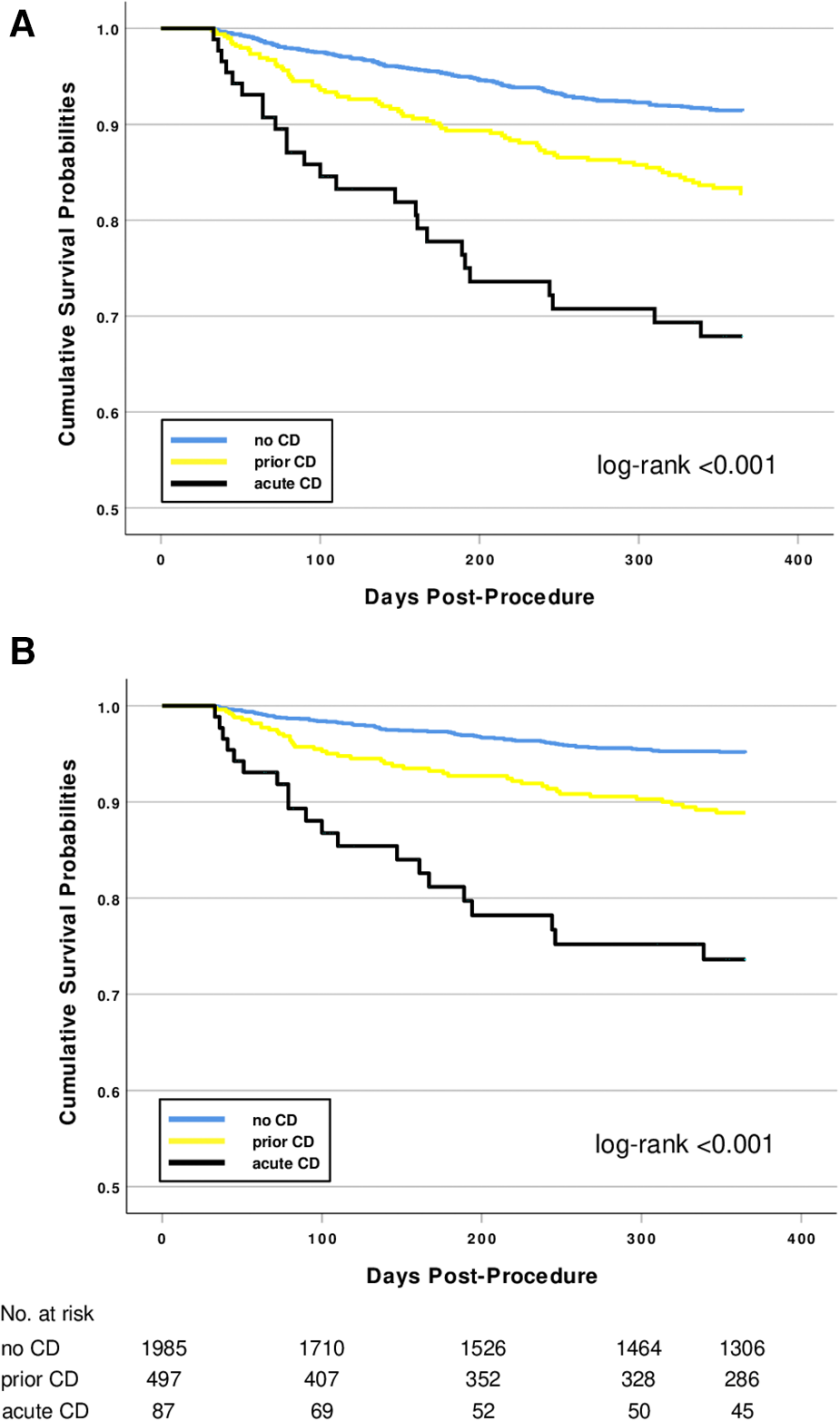
Kaplan–Meier survival curves after TAVI for patients with no, prior, or acute CD before hospital admission. (**A**) All-cause mortality. (**B**) Cardiovascular mortality.

One-year all-cause mortality rates for patients with no, prior, or acute CD were 7.4%, 14.5%, and 28.7% (log-rank <0.001), and cardiovascular mortality was 4.1%, 9.3%, and 23.0%, (log-rank *p* < 0.001, Figure 1); the event rates for the combination of cardiovascular death or new CD were 7.1%, 15.7% and 33.3% (*p* < 0.001). In the subgroup of patients with prior CD only, patients with 1, 2–6 months, or >6 months timespan between CD and TAVI had all-cause one-year mortality rates of 16.3%, 12.3%, and 11.4%, respectively (log-rank = 0.625). Cardiovascular one-year mortality rates were 10.0%, 6.5%, and 8.6%, respectively (log-rank = 0.573).

Univariate predictors of one-year all-cause mortality in the overall study population included parameters of body physique (low BMI, low PMAi), cardiovascular diseases (diabetes mellitus, atrial fibrillation, peripheral arterial disease, EuroScore II), clinical parameters (highly symptomatic patients in NYHA class III and IV, a reduced general condition, prior or acute CD, fluid overload), echocardiographic parameters (low ejection fraction, low transvalvular gradients, mitral or tricuspid valve regurgitation), laboratory parameters (anemia, renal insufficiency), and the absence of prescribed discharge medication (ACEI/ARB and statins). A summary of the results of the univariate Cox regression analysis is given in Table 3.

**Table 3:** Univariate Cox regression analysis with 1-year all-cause mortality as the outcome variable.

Variable	HR	CI 95%		*p*
		Lower	Upper	
Female	0.960	0.746	1.235	0.751
Age	1.024	1.000	1.049	0.053
BMI	0.954	0.928	0.981	0.001
COPD	1.051	0.764	1.445	0.760
Cardiovascular disease				
Arterial hypertension	0.627	0.433	0.910	0.014
Diabetes mellitus	1.527	1.183	1.970	0.001
CAD	1.031	0.796	1.335	0.817
Prior MI	0.703	0.454	1.090	0.115
Atrial fibrillation	2.450	1.889	3.176	<0.001
Prior stroke	1.176	0.825	1.675	0.371
Peripheral artery disease	1.567	1.120	2.192	0.009
EuroScore II	1.069	1.047	1.091	<0.001
ICD at discharge	1.690	0.897	3.182	0.104
Clinical data				
NYHA class III/IV	1.875	1.305	2.695	<0.001
General condition (Steps 1–3)				
- normal	–	–	–	–
- reduced	2.298	1.648	3.203	<0.001
- poor	5.055	2.809	9.099	<0.001
Frailty	1.262	0.977	1.631	0.075
Cardiac decompensation				
- no	–	–	–	–
- prior	2.102	1.585	2.788	<0.001
- acute	4.525	2.960	6.917	<0.001
Moist rales	2.192	1.414	3.398	<0.001
Peripheral oedema	1.870	1.420	2.462	<0.001
Fluid overload	2.534	1.950	3.293	<0.001
Laboratory Data				
GFR	0.989	.0985	0.994	<0.001
Anemia	1.895	1.470	2.444	<0.001
Echocardiographic data				
Ejection fraction	0.982	0.973	0.991	<0.001
Ejection fraction ≤40%	2.089	1.492	2.924	<0.001
MPG <40 mmHg	1.947	1.508	2.513	<0.001
Moderate MR or TR	1.649	1.234	2.204	<0.001
CT data				
Pleural effusion	2.909	2.211	3.826	<0.001
PMAi	0.903	0.846	0.963	0.002
PMAi tertiles (1–3)	0.816	0.695	0.958	0.013
Procedural data				
Balloon-expandable valve	0.853	0.654	1.113	0.241
Medication at hospital discharge				
ACEI/ARB	0.542	0.414	0.710	<0.001
Beta-blockers	1.423	1.032	1.961	0.031
Mineralocorticoid antagonists	1.273	0.935	1.734	0.126
Statins	0.626	0.484	0.810	<0.001

The following parameters emerged as independent predictors of one-year all-cause mortality in the multivariable analysis (Table 4): low BMI, diabetes mellitus, atrial fibrillation, a reduced general condition, prior or acute CD, fluid overload, anemia, a low-flow AS, and the lack of prescribed ACEI/ARB and of statins.

**Table 4:** Multivariate Cox regression analysis with 1-year all-cause mortality as the outcome variable.

Variable	HR	CI 95%		*p*
		Lower	Upper	
BMI	0.951	0.923	0.979	<0.001
Diabetes mellitus	1.434	1.074	1.014	0.015
Atrial fibrillation	1.756	1.322	2.334	<0.001
EuroScore II	1.035	1.007	1.063	0.015
General condition (Steps 1–3)				
- age-appropriate	reference	–	–	–
- reduced	1.529	1.062	2.202	0.022
- poor	1.201	0.575	2.509	0.627
Cardiac decompensation				
- no	reference	–	–	–
- prior	1.396	1.023	1.906	0.036
- acute	1.715	1.011	2.911	0.046
Fluid overload	1.537	1.136	2.079	0.005
Anemia	1.432	1.088	1.886	0.010
MPG <40 mmHg	1.436	1.084	1.902	0.012
ACEI/ARB	0.597	0.445	0.799	<0.001
Statins	0.659	0.498	0.872	0.003

HR, hazard ratio; CI, confidence interval; BMI, body mass index; MPG, mean pressure gradient; ACEI/ARB, angiotensin converting enzyme inhibitors or angiotensin receptor blocker.

**Figure 2:**
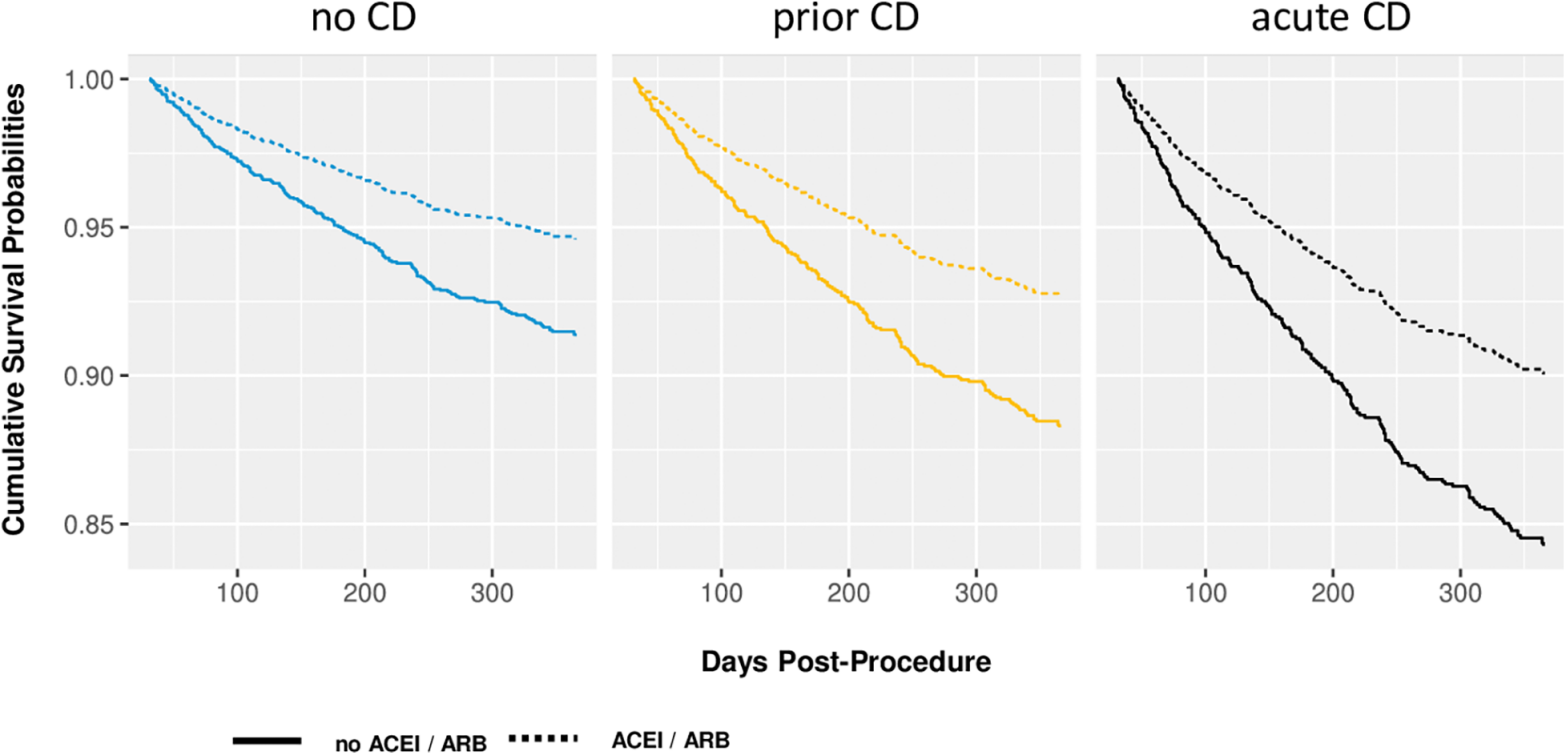
Adjusted survival curves illustrating the effect of both a history of cardiac decompensation and the effects of ACEI/ARB on one-year mortality. Predictions of the multivariate Cox proportional hazard model for prototypical values: BMI = 27.7 kg/m^2^, diabetes, atrial fibrillation, Euroscore II = 4.14%, reduced general condition, no fluid overload, no anemia, MPG >40 mmHg, statins.

Figure 2 illustrates the effect of prescribed ACEI/ARB medication on the hazards of one-year mortality on patients of all three CD classes. ACEI/ARB were associated with a reduction of mortality by around one third, and this effect was consistent throughout all three CD classes.

We finally analyzed patients with an EF ≤40% (*n* = 323) and those with an EF ≥50% (*n* = 2,121) in analogy to patients with heart failure with reduced or preserved EF, respectively (Table 5). Both groups revealed a significant increase in mortality rates from patients without CD over those with a prior to those with an acute CD. In patients with an EF ≤40%, prescribed ACEI/ARB therapy was associated with a numerically 50% reduction of mortality rates in patients with a prior and in those with an acute CD, however due to small patient numbers, the significance level was not achieved. In patients with an EF ≥50%, ACEI/ARB was associated with a significant survival benefit in patients with a prior CD while no effect could be observed in those patients with an acute CD.

**Table 5:** All-cause one-year mortality rates of patients with EF ≤40% vs. EF ≥50% .

Class of CD	ACEI/ARB	EF ≤40%	log-rank	EF ≥50%	log-rank
		*n* = 323		*n* = 2,121	
no CD		16/178 (9.0)	<0.001	124/1,720 (7.2)	<0.001
prior CD		21/108 (19.4)		45/355 (12.7)	
acute CD		13/37 (35.1)		10/46 (21.7)	
prior CD	yes	13/82 (15.9)	0.088	27/267 (10.1)	0.010
	no	8/26 (30.8)		18/87 (20.7)	
acute CD	yes	7/26 (26.9)	0.087	6/28 (21.4)	0.879
	no	6/11 (54.5)		4/18 (22.2)	

Data shown as number (%). ACEI/ARB, angiotensin converting enzyme inhibitors/angiogtensin receptor antagonists; CD, cardiac decompensation.

## Discussion

The classification of this all-comers population of patients with severe AS into those with no, prior, or acute CD and the thorough evaluation of signs of cardiac congestion and evaluation of their prognostic impact may lead to a better understanding of the prevalence and clinical manifestation of concomitant HF in patients with AS. While almost 90% of all patients presenting with acute CD had documented cardiac congestion, fluid overload was also manifest in more than 50% of the patients with prior CD and even in more than 25% of those patients with no reported CD, suggesting the presence of manifest or subclinical HF among the whole spectrum of patients with AS referred for TAVI.

We chose to include only patients fulfilling the criteria of 30-day device success, because we explicitly wanted to exclude the impact of procedural and technical aspects of TAVI that could influence further results, thereby attempting to investigate outcomes and the effect of the prescribed medication in apparently stable patients after successful TAVI. Our model allowed for a stepwise comparison of patient cohorts with increasing intensity of signs and symptoms of cardiac congestion, and it is intriguing to observe similarities to those of classic HF patients. Indeed, the loss of left ventricular systolic function as reflected by a reduction of ejection fraction and an increase in the frequency of low-gradient aortic stenosis, alongside with a higher prevalence of atrio-ventricular valve dysfunction, a functional decline of other organ systems manifested by a reduced renal function and anemia, and a clear accumulation of extravascular fluid in different body compartments are clearly compatible with signs of HF. However, even when our data suggest at first the presence of HF with reduced EF as the main origin of CD, more detailed analysis of our data revealed that CD-related mortality rates are similar in patients with reduced and preserved EF. This observation allows to conclude that mechanisms described in patients with HF with preserved EF are present in our patient population and that a part of those with an EF ≥50% might bear the (most probably masked) diagnosis of HF with preserved EF. Sarcopenia, though common in older patients, is a characteristic of patients with HF with a prevalence of around 34% (9), contributes to exercise intolerance and ventilatory inefficiency, and is associated with longer hospitalizations and worsened prognosis in these patients (10). Although we did not measure functional parameters of sarcopenia, it is interesting to observe an increased percentage of patients in the sex-specific lowest range of PMAi in the patient groups with prior and acute CD, which appears to be similar to patients with HF. Accordingly, more patients with prior or acute CD presented in a poor general clinical condition. Interestingly, each of the individual signs of volume overload (moist pulmonary rales, peripheral edema, pleural effusion) emerged as a strong univariate predictor of prognosis, and any fluid overload proved to be an independent prognostic indicator of one-year mortality.

Fluid overload, interpreted as residual clinical congestion, could be part of a pathophysiologic model that would explain the rather high mortality in the intermediate group of patients with prior CD. Of almost 8,000 patients with acute HF included in the ESC-EORP-HFA Heart Failure Long-Term Registry (11), 31% of those discharged alive from hospital still had signs of residual congestion, which is in line with the results of other studies (12), all of which demonstrated the strong negative predictive value of residual fluid overload in re-compensated HF patients for future re-hospitalizations or death (13). It seems plausible to decode the presence of fluid overload in patients with prior CD as a remnant of incomplete recompensation or subclinically manifested HF, presenting only the end results of fundamentally and persistently disrupted hemodynamics that obviously affect outcomes even after TAVI. Indeed, once symptomatic patients are diagnosed with severe AS, the aim of clinical management is for symptom improvement and improvement of prognosis; however, once the AS has been corrected by TAVI and symptoms have improved, the complete recompensation of patients might not be documented, and one could speculate that patients still have a lingering degree of cardiac congestion that is associated with higher rehospitalization rates and worse outcomes. Our analysis revealed that the shorter the timespan between CD and TAVI was, the more fluid overload in those patients was present, which could have translated into higher mortality rates in these patients with a very recent CD. However, no significant impact of the timespan on mortality could be observed. We speculate that this lacking relationship might be due to the different levels of cardiac recompensation that could be achieved during the hospital stay. Unfortunately, we cannot provide data on fluid overload at the timepoint of hospital discharge, information that might have contributed to a further understanding of the clinical status of HF in these patients.

From a clinical point of view, our results are consistent with the concept of an early treatment of patients with left ventricular dysfunction, even for those with a less-than-severe degree of aortic stenosis (14). The idea of this concept is that an early unloading of the left ventricle in these patients might avoid further deterioration of left ventricular function and avoid cardiac decompensations, a concept that is currently being tested in a multicenter study (15). In fact, one could conclude that an early treatment, most likely consisting of aortic valve replacement, should be initiated before signs of cardiac congestion are manifest.

The beneficial effect of targeted medication at hospital discharge has already been demonstrated for ACEI/ARB (8, 16) and statins (17) and demonstrates parallels to the effect in patients with HF. It is interesting to note that the ACEI/ARB regimen might be effective not only in stable patients without any prior CD but also in those with prior CD and even in those hospitalized for acute CD. Given the similarities to patients with HF with reduced EF, it is not surprising to see substantial effects of ACEI/ARB in these patients. However, it is interesting to note that significant effects of ACEI/ARB prescription could be observed in patients with probably masked HF with preserved EF who suffered a previous CD, even more because this medication has not been proven to be effective in this condition. Obviously additional/complementary pathophysiological mechanisms should play a role in patients with AS that make those patients amenable for the beneficial effects of ACEI/ARB.

Given that these results might hold true in prospective and controlled trials, such a therapy might comprise a potential treatment to deal with the excess mortality observed in patients with AS and HF after TAVI.

One limitation of this retrospective analysis may be the lack of a systemic, serial documentation of left ventricular function and fluid status during follow-up. Moreover, the knowledge of natriuretic peptide levels could have given important insights into myocardial wall stress. Regarding the prescribed medication of our patients, we cannot comment on true compliance during the observational period, and the prescribing of ACEI/ARB might have reflected a better overall clinical condition of those patients, translating into a better outcome *per se*.

Taken together, the results of our study help to better understand the clinical condition of patients with AS, in particular of those with or without a history of CD. In parallel to patients with HF, those patients of our registry with documented fluid overload had a worse prognosis compared with those without. The classification into acute, prior, or no CD additionally determined prognosis. These results may contribute to the discussion about an early treatment strategy in patients with AS and left ventricular dysfunction. Moreover, medical treatment post-TAVI might be a crucial part of the postinterventional treatment.

## Data Availability

The datasets presented in this article are not readily available because according to local restrictions no patient data, even anonymized, can be shared with other people outside our institution. Requests to access the datasets should be directed to www.kerckhoff-klinik.de.
